# Case Report: A rare synchronous multiple gastric carcinoma achieved progression-free disease through NGS-guided serial treatment

**DOI:** 10.3389/fonc.2023.1195837

**Published:** 2023-07-11

**Authors:** Xinyi Shao, Jin Yin, Di Wang, Erjiong Huang, Yini Zhang, Jiani C. Yin, Chen Huang, Hao Wu, Xiaoli Wu

**Affiliations:** ^1^ Department of Gastroenterology, The First Affiliated Hospital of Wenzhou Medical University, Wenzhou, Zhejiang, China; ^2^ Department of Medicine, Nanjing Geneseeq Technology Inc., Nanjing, Jiangsu, China

**Keywords:** synchronous multiple gastric carcinoma (SMGC), next-generation sequencing (NGS), circulating tumor DNA (ctDNA), immunotherapy, anti-HER2 treatment

## Abstract

Synchronous multiple gastric carcinoma (SMGC) is a rare condition characterized by the simultaneous occurrence of two or more primary malignant tumors in the stomach, each with its own distinct pathological morphology. SMGC differs from gastric metastases, which originate from primary gastric or non-gastric tumors. At present, the incidence of SMGC is low in China, with no established guidelines for standard treatment. Here, we report a rare case of advanced SMGC that achieved long-lasting clinical benefits through a treatment strategy informed by next-generation sequencing (NGS). Dynamically monitoring of the tumor and/or circulating cell-free DNA guided the patient’s treatment sequentially. The patient received anti-HER2 therapy, followed by immunotherapy, pembrolizumab in combination with trastuzumab and chemotherapy, and ultimately underwent successful total gastrectomy. This case highlights a novel approach of utilizing liquid biopsy-based NGS to gain insights into disease progression and molecular response to NGS-guided treatment in SMGC patients.

## Introduction

Gastric cancer ranks as the fifth most prevalent cancer and the fourth leading cause of cancer mortality globally (1). Synchronous multiple primary gastric cancer (SMGC) is characterized by the simultaneous development of two or more primary malignant tumors in the stomach, which must be distinguished from metastatic lesions that originate from the same site (2). Currently, the incidence of SMGC in China is relatively low, accounting for only 1%-5% of gastric cancers, although it is gradually increasing (3). Compared to solitary gastric cancer, SMGC is more commonly observed in elderly male patients and is frequently found in the upper stomach (4). It has been reported that the prognosis of SMGC is comparable to that of single gastric cancers, with a postoperative five-year survival rate of ≥90% (5). Both the TNM stage and the primary tumor T stage are associated with postoperative survival in SMGC patients (6).

Currently, the main therapeutic modalities for gastric cancer include chemotherapy, targeted therapies, and immune checkpoint inhibitors. While chemotherapy has been well-established as a standard treatment option (7, 8), the advent of targeted therapies and immunotherapies has greatly expanded the therapeutic landscape for gastric cancer and numerous other malignancies. Molecular-targeted agents, such as nivolumab (anti-PD-1), pembrolizumab (anti-PD-1), ramucirumab (anti-VEGFR2), and trastuzumab (anti-HER2), have been approved for the treatment of gastric cancer (9). The molecular classification of gastric cancer is based on the expression of human epidermal growth factor receptor 2 (HER-2) in tumor tissues, which serves as the basis for selecting anti-HER2 targeted therapies (10). On the other hand, immune checkpoint inhibitors (ICIs) targeting programmed death protein-1 (PD-1) and its ligand-1 (PD-L1) have become a focus of research in tumor immunotherapy in recent years (11, 12). Despite being an effective treatment for cancer, ICIs only lead to durable responses in a subset of unselected patients, especially when used as a single-agent therapy (13, 14). The identification of predictive biomarkers, such as tumor mutational burden (TMB), PD-L1 expression, and microsatellite instability (MSI)/deficient mismatch repair (dMMR), has enabled the enrichment of potential responders to ICIs. Next-generation sequencing (NGS) has played a significant role in deepening our understanding of the genomic architecture of various cancers and uncovering potential therapeutic targets for personalized treatment (15). However, the use of NGS in gastric cancer is still in its investigational phase.

Given that the multiple primary tumors in SMGCs originate from distinct sources, the mutational profiles of each tumor differ significantly. Consequently, an integrative treatment approach that takes into consideration the differences in pathological features and genetic constitutions of each tumor is necessary. In this case report, we present a unique case of SMGC that successfully underwent a complex treatment sequence, directed guided by dynamic monitoring of genetic mutations. The patient achieved an overall survival of more than 43 months at the time of manuscript preparation.

## Case presentation

**Figure 1:**
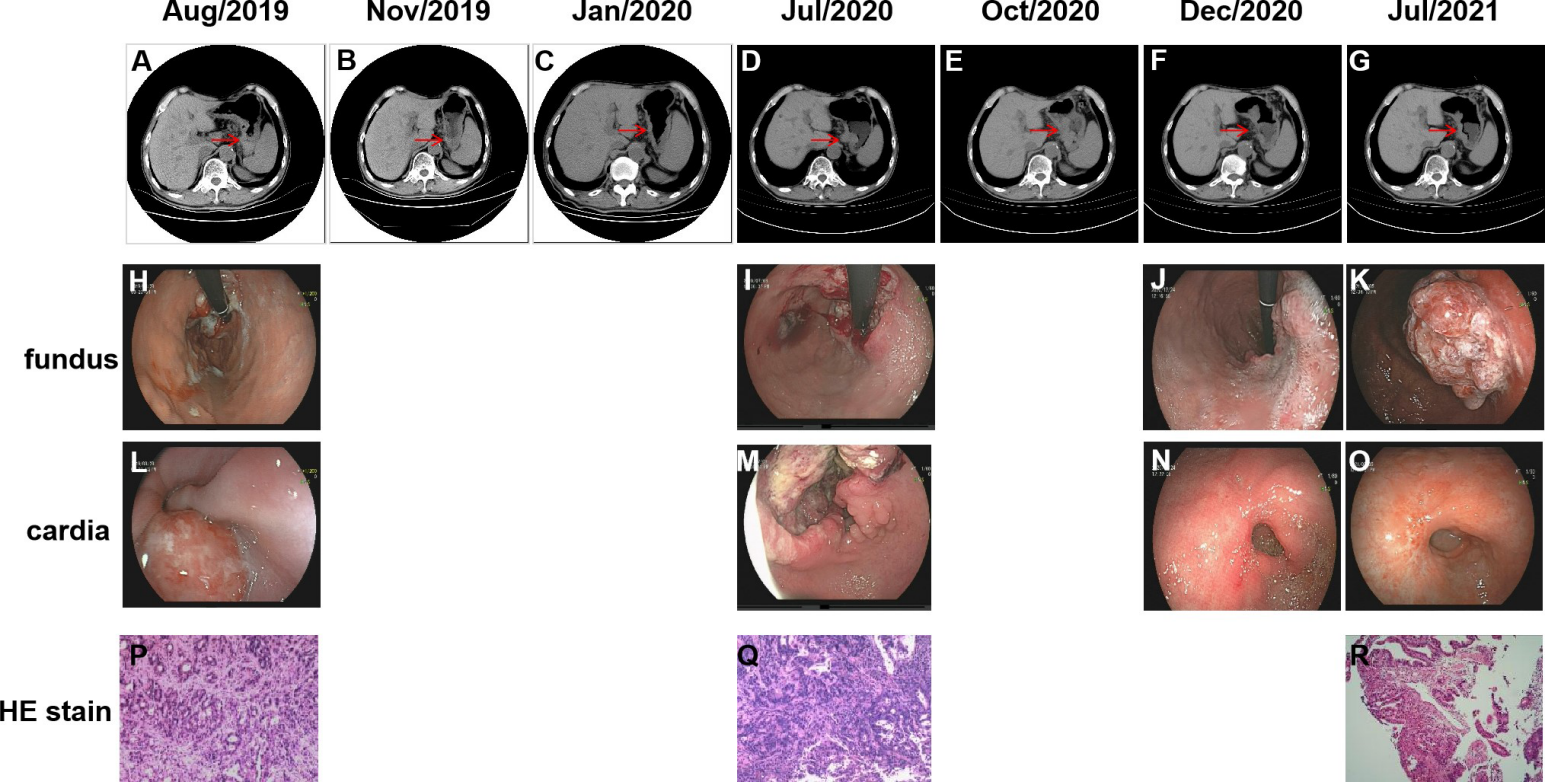
Abdominal CT images and gastroscopic images of the two independent lesions during the course of the treatment. Gastroscopic images of the two independent lesions from cardia **(L–O)** and fundus **(H–K)** are shown as well as the HE stain **(P–R)**. Lesions in abdominal CT images **(A–G)** during treatment are indicated by the red arrows.

**Figure 2:**
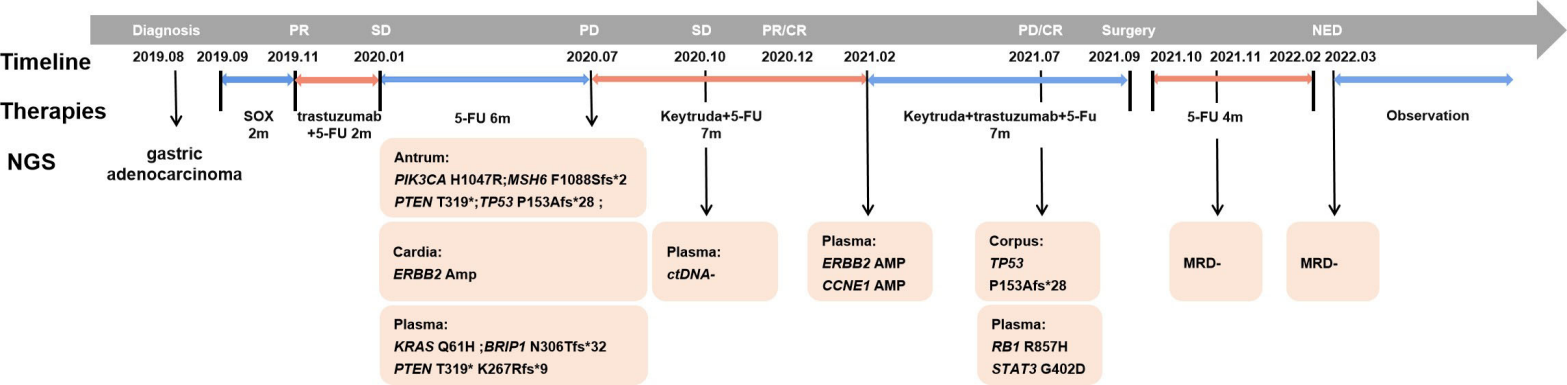
Diagnosis and treatment process of the presented case. The medical history of this SMGC case is shown with genetic information, treatment schedule, timeline and response evaluation. Plasma ctDNA sequencing was performed along with treatment response evaluation as indicated during the sequential treatment.

A 75-year-old Chinese male was admitted to our hospital in August 2019, with symptoms of recurrent abdominal pain and dysphagia lasting for three weeks. CT scans revealed thickening of the gastric wall in the fundus, cardia and body regions (**Figure 1A**), along with the presence of multiple lymph nodes in the hepatogastric ligament region and posterior pancreatic nodules suggestive of tumor metastasis. Further, Positron Emission Tomography-Computed Tomography (PET-CT) indicated malignancies in the gastric cardia and the inferior and posterior gastric walls, accompanied by multiple lymph node metastases. Gastroscopy revealed multiple ulcerative lesions throughout the stomach, with irregular elevation of the surrounding mucosa (**Figures 1H, L**). Gastroscopic biopsy and immunohistochemical (IHC) staining of the cardia confirmed the presence of adenocarcinoma (**Figure 1P**), with strong positive expression of HER-2 (HER-2+++). Subsequently, the patient was diagnosed with gastric adenocarcinoma (cT4N3bM0, stage IIIc) (**Figure 2**). In September, due to the lack of motivation and personal preference, the patient was treated with first-line chemotherapy, consisting of oxaliplatin (Eloxatin) injection at a dose of 200 mg on Day 1, and Tegio capsules at a dose of 40 mg orally, twice daily from Day 1 to Day 14, with treatment cycles repeated every three weeks for a maximum of three cycles. By November, the disease achieved partial response (PR) according to imaging assessments (**Figure 1B**). With the perceived medical benefits, the patient’s treatment approach was modified to a combination of chemotherapy and anti-HER2 targeted therapy for HER2-positive gastric cancer. The regimen involved Trastuzumab injections and Tegio capsules administered every three weeks for a maximum of three cycles. However, the patient developed intolerant symptoms shortly after the initiation of the targeted treatment, leading to its discontinuation until Jan 2020, when the disease remained stable by a CT scan (**Figure 1C**). Following six cycles of chemotherapy, the patient was transitioned to long-term maintenance with oral Tegio capsules and scheduled for regular followed-up visits at the clinic. Unfortunately, the disease progressed rapidly within a span of two months, as evidenced by follow‐up CT scans. In July 2020, the patient was admitted to the hospital due to abdominal pain and emaciation, and was confirmed to have progressive disease (PD) by a CT scan (**Figure 1D**). Gastroscopy revealed two lesions in the cardia and antrum, with normal mucosa observed between the two regions (**Figures 1I, M**). Interestingly, while both lesions were diagnosed as adenocarcinoma (**Figure 1Q**), IHC staining showed HER2 positivity only in the cardia, and not in the antrum.

**Figure 3:**
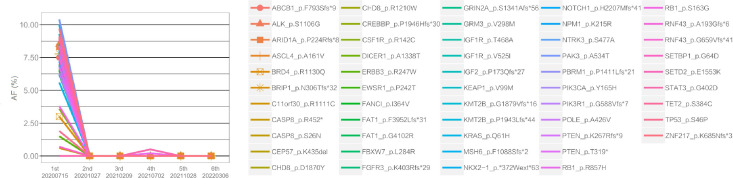
Changes in the allele frequencies (AFs) of mutations detected in serial plasma ctDNA during treatment. The X-axis represents the time points of plasma ctDNA sequencing, and the Y-axis indicates the AFs of different mutations, represented by color-coded lines. As shown in the figure, the AF levels of nearly all mutations exhibited varying degrees of decline following treatment.

To determine whether the two lesions were synchronous primaries or metastases, we performed NGS on the biopsy samples from both the cardia and antrum, as well as plasma samples using a 425 cancer-relevant gene panel, GeneseeqPrime®, at a CLIA-certified and CAP-accredited laboratory (Nanjing Geneseeq Technology, Jiangsu, China). Notably, no overlapping mutations were detected between the lesions in the cardia and antrum (**Supplementary Table S1**). Consistent with the IHC results, *ERBB2* amplification was only detected in the cardia lesion, and not in the antrum lesion. Based on these results, we inferred that the lesions represented two primary tumors arising from different origins. In addition, we detected *KRAS* p.Q61H and *PTEN* p.K267Rfs*9 in the plasma. Although not detected in the tissue samples, the presence of these two variants is likely to explain the relatively poor response to anti-HER2 inhibitors. Both the antrum and cardia lesions exhibited hyper-mutation, with tumor mutational burden (TMB) of 82.7 and 18.3 mut/Mb, respectively. The antrum lesion also displayed microsatellite instability, likely attributed to a frameshift mutation in the MutS Homolog 6 (*MSH6*) gene. Consequently, the patient’s treatment was modified to a combination of pembrolizumab and chemotherapy. NGS profiling at three months after initiating treatment demonstrated a substantial decrease in circulating tumor DNA (ctDNA) levels (**Figure 3**), which was consistent with stable disease (SD) of the lesions (**Figure 1E**). At five months following chemo-immune combination therapy, the antrum lesion achieved complete response (CR), the cardia and body lesions achieved partial response (PR) as evaluated by CT scans and Gastroscopy (**Figures 1F, J, N**).

However, disease progressed again after seven months of immunotherapy. To identify an alternative therapeutic option, ctDNA-based NGS was once again performed. As shown in **Supplementary Table S1**, *ERBB2* and *CCNE1* amplifications were detected at copy numbers of 3.5 and 3.6, respectively. Subsequently, trastuzumab was re-introduced to the treatment regimen in February 2021. The therapy was continued for five months until July 2021, when the patient presented with a new gastric body mass (**Figures 1G, K, O, R**), and ctDNA was detected in the plasma (**Figure 2**). Compared to the previously analyzed lesions, the mutational profile of the newly developed body mass was highly consistent with that of the cardia lesion, suggesting metastasis. In August 2021, a PET-CT scan revealed a decrease in hyper-metabolism of the small lymph nodes in the hilum and perigastric regions compared to previous scans, suggesting regression of the metastatic tumor and potential resectability. In September 2021, the patient successfully underwent a total gastrectomy. Starting from October 2021, the patient received adjuvant therapy with oral Tegio capsules for four months. Two months after the surgery, CT scans showed no visible lesions. Furthermore, NGS analysis of the plasma ctDNA confirmed the absence of molecular residual disease. The patient is currently being closely monitored during the follow-up period. Remarkably, as of the time of submission, the patient has achieved a disease-free status for 17 months, with an overall survival of 43 months since the initial diagnosis.

## Discussion

In this report, we described a case of unresectable SMGC that underwent a complex treatment sequence guided by dynamic monitoring of genetic mutations, ultimately leading to successful surgery. Notably, the patient reached an OS exceeding 43 months at the last follow-up.

The incidence of SMGC has been increasing in recent years due to advancements in diagnostic approaches and more accurate pathological examinations. It is crucial to pay more attention during pre- and intra-operative examinations to avoid missed diagnoses (16). Multiple clinical factors have been identified to be predictive of SMGC outcome. In addition to main tumor T stage and TNM stage, lymph node metastasis, preoperative AFP levels and nerve invasion may also be associated with SMGC recurrence (6). Furthermore, the level of preoperative CA125 and lymphovascular cancer plug have been identified as independent risk factors for lymph node metastasis in SMGC patients (17). Total tumor volume (TTV) may also serve as a prognostic factor in SMGC with curative gastrectomy (18). Clinical evaluation should be conducted meticulously to improve the accuracy of prognostic prediction in SMGC.

The development of targeted therapies and immunotherapy has greatly improved outcomes for patients with advanced gastric cancer (19, 20), which largely rely on our understanding of its molecular profiles. However, in the case of SMGC, the presence of multiple primary tumors implies distinct molecular origins. Indeed, we observed non-overlapping genomic profiles between the two primary lesions in our case. *ERBB2* amplification was detected in only one of the lesions, consistent with IHC results. The lack of a shared driver alteration may explain the limited effectiveness of anti-HER2 therapy in our patient. Despite the different molecular landscape, both lesions exhibited a high TMB. As a surrogate for neoantigen (21), numerous studies have shown that TMB is associated with the efficacy of anti-PD-1 or PD-L1 inhibitors in diverse tumors (22). The CheckMate 649 study has found that the nivolumab and chemotherapy combination is associated with a better overall survival benefit than chemotherapy alone, with patients exhibiting high TMB deriving a higher degree of survival benefit (23). In our case, all lesions exhibited durable responses to the combination of pembrolizumab and chemotherapy.

The presence of minimal residual disease (MRD) has been demonstrated as a significant predictor of recurrence and prognosis in gastrointestinal cancer patients (24–28). Biomarkers such as *HER2*, *KRAS* and *BRAF* have been utilized in gastric cancer patients to detect MRD. In our case, liquid biopsy-based NGS allowed for monitoring of treatment outcomes through fluctuating ctDNA levels that correlated with response. We detected *ERBB2* amplification in the ctDNA at disease progression seven months after the immune-chemotherapy combination. By re-introducing trastuzumab into the treatment regimen, the patient achieved molecular remission once again. Furthermore, the patient exhibited negative MRD as indicated by a clearance of ctDNA after surgical resection. These results are consistent with the patient’s long-term benefit, with a DFS of over 17 months and an OS of over 43 months. Our findings highlights the crucial value of ctDNA serial monitoring to gain deeper insight into treatment response and risk of recurrence over time.

## Conclusion

We hereby reported a rare case of SMGC that achieved long-lasting benefits through NGS-guided treatment, resulting in an OS exceeding 43 months. Our case, along with other works, underscores the clinical utility of liquid-based NGS, particularly in the dynamic monitoring of disease outcomes. Our case also highlights the significance of NGS in SMGC for accurately differentiating multiple primary tumors and metastases, facilitating informed therapeutic decisions, and allowing for timely adjustments to treatment regimens during the course of clinical care.

## Data availability statement

The datasets presented in this study can be found in online repositories. The names of the repository/repositories and accession number(s) can be found in the article/**Supplementary Material**.

## Ethics statement

The study involving human participant was approved by the committee of the First Affiliated Hospital of WenZhou Medical University and was conducted in accordance with the Declaration of Helsinki. Written informed consent was obtained from the patient for publication of this case report and any accompanying images.

## Author contributions

XW involved in conception and design. XS and JY carried out provision of study material or patients. EH, YZ, HW and CH interpreted the data. DW and JCY provided NGS technical support. XS, HW and JY wrote the manuscript. All authors contributed to the article and approved the submitted version.
